# Advances in Understanding the Antioxidant and Antigenic Properties of Egg-Derived Peptides

**DOI:** 10.3390/molecules29061327

**Published:** 2024-03-16

**Authors:** Mihaela Brumă (Călin), Ina Vasilean, Leontina Grigore-Gurgu, Iuliana Banu, Iuliana Aprodu

**Affiliations:** Faculty of Food Science and Engineering, Dunarea de Jos University of Galati, 111 Domneasca Street, 800201 Galati, Romania; mihaela.calin@ugal.ro (M.B.); ina.vasilean@ugal.ro (I.V.); leontina.gurgu@ugal.ro (L.G.-G.); iuliana.banu@ugal.ro (I.B.)

**Keywords:** egg proteins, proteolytic enzymes, hydrolysis degree, antioxidant activity, antigenic properties

## Abstract

Pepsin, trypsin and proteinase K were used in the present study to hydrolyse the proteins from whole eggs, yolks or whites, and the resulting hydrolysates were characterised in terms of antioxidant and IgE-binding properties, using a combination of in vitro and in silico methods. Based on the degree of hydrolysis (DH) results, the egg yolk proteins are better substrates for all the tested enzymes (DH of 6.2–20.1%) compared to those from egg whites (DH of 2.0–4.4%). The SDS-PAGE analysis indicated that pepsin and proteinase K were more efficient compared to trypsin in breaking the intramolecular peptide bonds of the high molecular weight egg proteins. For all the tested substrates, enzyme-assisted hydrolysis resulted in a significant increase in antioxidant activity, suggesting that many bioactive peptides are encrypted in inactive forms in the parent proteins. The hydrolysates obtained with proteinase K exhibited the highest DPPH radical scavenging activity (124–311 µM Trolox/g protein) and the lowest residual IgE-binding capacity. The bioinformatics tools revealed that proteinase K is able to break the integrity of the main linear IgE-binding epitopes from ovalbumin and ovomucoid. It can be concluded that proteinase K is a promising tool for modulating the intrinsic properties of egg proteins.

## 1. Introduction

Chicken eggs have an undisputedly high biological value, being a good source of various macro- and micro-nutrients. The high nutritional value of the eggs is due to the high content of proteins with an ideal amino acids profile, essential lipids, vitamins and minerals [1,2]. These components are not evenly distributed between the egg components: the egg yolk consists of 55.00% water, 26.70% fats, 15.50% proteins, 1.68% ash and 1.09% carbohydrates, while the egg white has significantly higher amounts of water (87.60%) and significantly lower contents of proteins (10.80%), fats (0.19%), ash (0.62%) and carbohydrates (0.85%) [3].

Fractionation of the egg white and yolk is one efficient way to limit the egg consumption decline caused by the high cholesterol or fat content [4]. Because of the important differences regarding the composition of the two egg components, their techno-functional properties are very different. Properties like gel-forming ability, emulsifying activity, foaming capacity, colouring or flavouring properties are decisive in deciding their use for obtaining various food products, such as baked goods, mayonnaise, dressings, soufflés, meringues, etc. [5,6,7].

In addition to their high nutritional value and good functional properties, eggs have captured the interest of many researchers because of their health-related benefits, associated to anticancer, immunomodulatory, antioxidant, antihypertensive, antiviral and antimicrobial properties [4,7,8]. The accumulation of free radicals in organisms was associated with important oxidative damage of biologically important macromolecules, such as proteins and DNA, being also related to multifactorial diseases like cardiovascular disorders, cancer and diabetes [8]. Therefore, a diet rich in compounds with antioxidant activity is important for maintaining human health. Because of some properties like specificity, selectivity and potency, the bioactive peptides resulting from enzyme-assisted hydrolysis from food proteins are highly desired as an alternative to the usual small molecule chemotherapeutics [8]. Indeed, an increasing interest in developing nutraceuticals and functional foods with enhanced physiological functionality, due to the presence of bioactive peptides, was observed.

There are many studies showing that regular egg consumption might ensure health benefits to humans, but the allergies induced by egg proteins should not be neglected. In fact, chicken eggs are among the most common allergic foods, with increasing incidences in the last years [5]. Proteins from both egg components are reported to cause mild-to-severe allergic reactions, especially in cases of children and infants, the main allergens being ovomucoid (Gal d 1), ovalbumin (Gal d 2), ovotransferrin (Gal d 3) and lysozyme (Gal d 4) from the egg white, α-livetin (Gal d 5) and glycoprotein YGP42 (Gal d 6) from the egg yolk [4,5,8,9]. The concerns regarding the allergic responses associated with egg consumption limit their wide use as an ingredient in different food products. Considering that the threshold level for inducing adverse reactions to eggs varies in a large range (0.2–200 mg protein) for different individuals [10,11], it is crucial to identify suitable treatments for destroying the linear and/or conformational epitopes recognised by the IgE. Several attempts were made to lower the allergenicity of egg proteins by employing various processing technologies, involving enzyme-assisted hydrolysis, thermal treatment, high-pressure treatment, etc. [4]. In this respect, proteases were widely recognised as an important biotechnological tool for modulating the functional properties and biological activities of egg proteins to provide therapeutic benefits for human health, in addition to nutritional ones [4,12]. Therefore, identifying enzyme processing that ensures obtaining better intrinsic properties of the egg proteins, while improving the techno-functional properties became an important challenge.

Although limited hydrolysis improves functional properties, extensive hydrolysis usually allows lower residual antigenicity. Taking into account that eggs are commonly processed either as a whole or separately as yolks or whites, the aim of the study was to evaluate the effect of extensive enzyme-assisted hydrolysis on the properties of proteins originating from different egg components. The egg white, egg yolk, and whole egg were subjected to digestion with pepsin, trypsin and proteinase K, and the antioxidant and antigenic properties of the hydrolysates were determined by combining the in vitro and in silico approaches. Many studies dealing with the effect of gastric or intestinal digestion on chicken egg proteins are available in the literature, but no information regarding the impact of proteinase K is available. The efficiency of the microbial proteinase K to release peptides with reduced IgE-binding capacity was tested to check the possibility of using it on a larger scale for producing safer egg-based ingredients with improved functionality.

## 2. Results

### 2.1. Influence of the Enzyme on Egg Protein Hydrolysis

**Figure 1:**
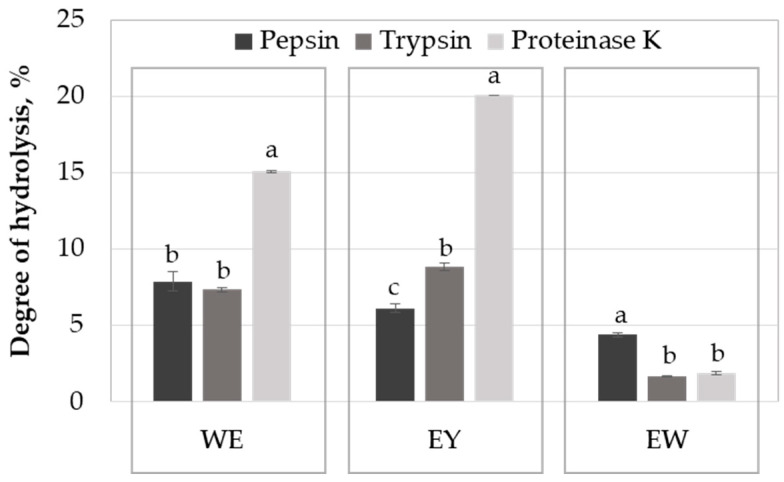
Comparison of the degree of hydrolysis (DH) of the whole egg (WE), egg yolk (EY) and egg white (EW) after 24 h of assisted hydrolysis with pepsin, trypsin and proteinase K. For an egg derivative (WE, EY and EW), different letters (a, b, c) assigned to the mean values indicate significant differences among enzyme treatments, at *p* < 0.05, based on Tukey post-hoc test and 95% confidence.

The degree of hydrolysis (DH) of the proteins from a whole egg (WE), egg yolk (EY) and egg white (EW) with pepsin, trypsin and proteinase K was determined using the pH-stat method after 24 h of reaction time (Figure 1). When using the whole egg as a substrate, the DH ranged between 7.3 and 15.1%; proteinase K was the most efficient in breaking the peptide bonds of the egg proteins, while no important differences were observed regarding the efficiency of the digestive enzymes. 

Among all the investigated samples, the egg yolk proteins allowed for obtaining the highest DH values. Egg yolk proteins are good substrates for proteinase K (DH of 20.1%), followed by trypsin (DH of 8.8%) and pepsin (DH of 6.2%). Proteinase K is a non-specific serine endopeptidase, recognised for its high proteolytic activity [13], explaining the high DH values registered when acting on WE and EY samples (Figure 1). The lower solubility of the proteins at acidic pH specific to the gastric environment, where perturbation of the specific protein folding and denaturation events occur [14], might explain the reduced hydrolysis degrees noticed in the case of pepsin. 

Regarding the egg white proteins, the highest DH of about 4.4% was registered for the sample prepared with pepsin, whereas no significant difference between samples prepared with trypsin and proteinase K was observed (Figure 1). The significantly lower DH values registered when using the egg white proteins as the substrate can be explained by the existence of important amounts of protease inhibitors. Ovomucoid is the main trypsin inhibitor found in hen egg white, while the ovoinhibitor has a wider inhibitory spectrum, being active against a larger number of proteases, including proteinase K, which is a serine-protease [8,15]. Both ovomucoid and ovoinhibitor belong to the Kazal family of proteinase inhibitors, but the egg white also hosts ovostatin with inhibitory activity towards all mechanistic proteinase classes, and cystatin, which is active against cysteine proteinases [15]. In addition to the antitypic activity, ovostatin is particularly active against metalloproteinases. Saxena and Tayyab [15] reported that, upon the formation of the proteinase–ovostatin complex, the approaching of the large molecule substrate to the active site is sterically hindered.

The susceptibility of egg proteins to be hydrolysed by various proteases, including those acting in the gastrointestinal tract, is affected in different manners by the applied processing technologies, which induce various conformational changes, altering the accessibility of the enzymes to the cleavage sites [7]. On the other hand, the impact of processing on the trypsin inhibitor activity should be also considered. Processing techniques involving high-temperature values have a special position when dealing with eggs, being often employed for cooking the eggs, stabilising the egg products, inactivating the microorganisms and enzymes and improving the sensory attributes and nutritional properties of the products [7]. Although no thermal treatment was applied to the protein derivatives used in the present study prior to enzyme addition, the technology applied for obtaining the powders typically involves processing at temperatures of 60–68 °C for 2–10 min for the pasteurisation effect, followed by spray drying at an inlet and outlet air temperature of 165–195 °C and 60–80 °C, respectively [7,16]. Several studies aimed at identifying the effect of processing on the digestibility of egg proteins. In this respect, several static and dynamic in vitro models were applied to mimic human gastrointestinal digestion [7]. The egg white proteins are generally thermolabile, and the preliminary thermal treatment applied to this substrate has a high influence on the digestibility of different proteins. Moreover, the susceptibility of different egg proteins to hydrolysis depends on their structural features and aggregation behaviour and varies with the peptidase used [14].

Lechevalier et al. [17] investigated the impact of whole-egg pasteurisation on the proteins’ digestibility and antigenicity. Regardless of the pasteurisation regime, they reported significantly higher DH values in the case of intestinal digestion, with respect to the gastric one. The differences between this trend and our results, indicating similar DH values for the whole egg protein hydrolysates obtained with pepsin and trypsin (Figure 1), might be explained by the fact that Lechevalier et al. [17] predigested the proteins with pepsin, therefore exposing the new hydrolysis sites to a higher extent, before performing the intestinal digestion using a simulated duodenal fluid with two different enzymes, trypsin and α-chymotrypsin, which have different specificity for the protein substrate. Our results highlight the importance of efficient gastric digestion of the egg proteins, prior to advancing towards the next segment of the digestive tract. In order to prevent the potential allergenicity issues associated with the low pepsin activity in individuals with incomplete maturation of the stomach function, it is therefore important to predigest the egg proteins by means of the exogenous enzymes prior to their use as ingredients in different food products.

Regarding the egg white proteins, the digestion with pepsin of the ovalbumin and ovomucoid in the raw samples was limited but the hydrolysis result was improved in the case of the heat-coagulated egg white [18]. In their native state, ovalbumin and lysozyme are more resistant to hydrolysis with pepsin compared to ovotransferrin, whereas the susceptibility of all three egg white proteins to hydrolysis with trypsin is limited. Liu et al. [14] reported that heat treatment of egg white proteins at 80 °C resulted in significant improvement in their susceptibility to hydrolysis with pepsin. Heating at 60 °C allowed substantial hydrolysis during the gastric digestion of the ovotransferrin and lysozyme, unlike ovalbumin, which was not efficiently attacked by the pepsin [14]. On the other hand, the authors reported the gradual hydrolysis of the ovalbumin during intestinal digestion. Owing to the more profound thermally induced protein structural changes accompanied by the exposure of the hydrolysis site, raising the temperature up to 80 °C allowed instead for the complete hydrolysis of ovalbumin, ovotransferrin and lysozyme by pepsin [14]. Of particular importance is that the thermal treatment affects the inhibitory trypsin activity exerted by ovomucoid. A kinetic study performed by van der Planken et al. [19] indicated a time-dependent decrease in the antitrypsin activity of ovomucoid when heated in the temperature domain of 75–100 °C. They indicated that the thermal stability of the ovomucoid is lower under particular conditions resembling the fresh egg white medium in terms of pH (minimum thermal stability at pH of 7.6) and the presence of other constituents, such as lysozyme.

### 2.2. SDS-PAGE Analysis of Egg Protein Hydrolysates

**Figure 2:**
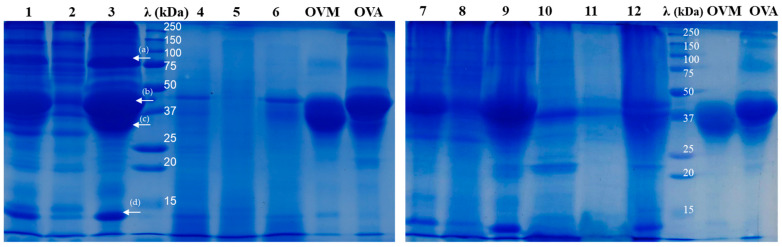
The SDS-PAGE profile of a whole egg, egg yolk and egg white after 24 h of assisted hydrolysis with pepsin, trypsin and proteinase K. Lanes 1, 2 and 3—the patterns of undigested whole egg (WE), egg yolk (EY) and egg white (EW), respectively; λ—Dual Xtra Standard (Bio-Rad, Hercules, CA, USA); lanes 4, 5 and 6—WE, EY and EW digested with pepsin; lanes 7, 8 and 9—WE, EY and EW digested with trypsin; lanes 10, 11 and 12—WE, EY and EW digested with proteinase K; OVM—the profile of the commercial ovomucoid; OVA—the profile of the commercial ovalbumin. The arrows on lane 3 are used to mark the main egg white proteins: (a) ovotransferrin (MW of ~78 kDa; [24]), (b) ovalbumin (MW of ~43 kDa [24], comparable with the pattern developed by the commercial OVA), (c) ovomucoid (MW of 30–40 kDa [25] matching the pattern of the commercial OVM) and (d) lysozyme (MW of 14.3 kDa, [24]).

The SDS-PAGE analysis under reducing conditions was applied to compare the profile of the protein hydrolysates generated with pepsin, trypsin and proteinase K, while using the proteins from the powdered whole egg or the separated components (egg white and yolk) as substrates. Analysing the results presented in Figure 1 and Figure 2, one can see that not only the hydrolysis degree varied with the enzyme used but also the peptide molecular size distribution of the resulting hydrolysates. 

Among all the available literature on egg white and egg yolk, Sarantidi et al. [2] recently reported the highest number of proteins, 398 and 456, respectively, identified through an LC–MS/MS intrinsic design. The most abundant proteins found in egg white are ovalbumin (54.0%), followed by ovotransferrin (12.0–14.0%), ovomucoid (11.0%), ovomucin (3.5%) and lysozyme (3.4–3.5%), while few other minor proteins, such as ovoinhibitor, ovoglycoprotein, ovoflavoprotein, ovomacroglobulin (also known as ovostatin), cystatin and avidin, are present in low percentages of 0.05–1.5% [1,4,20,21]. The molecular weight (MW) of egg white proteins ranges from 12.7 kDa, corresponding to the cystatin, to 8000 kDa, corresponding to the soluble ovomucin [8]. Egg yolk contains hundreds of proteins identified in the plasma fraction, and just 86 in the granular fraction [22], with molecular weights ranging from 5 to 200 kDa [23], the most abundant ones with important biological functions being the ones that are mainly described. The major proteins found in egg yolk are the low-density lipoproteins (68%), high-density lipoproteins (16%), livetins and other soluble proteins (10%) and phosvitin (4%) [1,7,20].

Figure 2 depicts the protein patterns of the whole egg (lane 1), egg yolk (lane 2) and egg white (lane 3) used in present the study, together with two major egg white proteins with their demonstrated allergenic potential, namely OVM and OVA.

In the case of all the investigated egg-based protein substrates, the hydrolysis with pepsin (lanes 4, 5 and 6 in Figure 2) resulted in an important reduction of the bands intensity associated with proteins with an MW higher than 28 kDa, compared to the corresponding undigested samples (lanes 1, 2 and 3, respectively, in Figure 2). These results suggest that pepsin was able to hydrolyse the most abundant and high MW proteins found in the egg-based samples. It can be also observed that OVM from the WE and EW samples was extensively hydrolysed by pepsin, resulting in peptides with lower MW. Our results comply with the observations of Abeyrathne et al. [25], who reported the almost complete hydrolysis of ovomucoid with 1% pepsin after 24 h of incubation at 37 °C, resulting in peptides with MW of 2–10 kDa. Analysing the bands intensity in lanes 4 and 6 (Figure 2), one can observe the existence of higher amounts of unhydrolysed OVA (MW of 43 kDa, Lassé’ et al. [24]) compared to OVM. Moreover, considering that the band at 78kDa noticed in the WE and the EW samples (lanes 1 and 3 in Figure 2) disappeared upon hydrolysis with pepsin (lanes 4 and 6 in Figure 2), we can assume that the enzyme ensured the complete hydrolysis of ovotransferrin in the case of both samples. These observations are in agreement with the results reported by Liu et al. [14], regarding the substantial pepsin-assisted hydrolysis of ovotransferrin from the heat-treated egg white samples.

The electrophoresis results indicated that pepsin was more efficient than trypsin in reducing the MW of the major egg proteins. When trypsin was used in the digestion process, the bands in lanes 7 (WE-T) and 9 (EW-T), corresponding to OVM and OVA, indicate that these allergen proteins were resistant to trypsin. In agreement with the hydrolysis degree results (Figure 1), because of the existence of trypsin inhibitors, the intensity of the bands corresponding to proteins with an MW over 28 kDa was higher in the hydrolysates obtained from the WE (lane 7) and the EW (lane 9), compared to the EY (lane 8) (Figure 2).

In agreement with the DH results presented in Figure 1, proteinase K was the most effective in hydrolysing the EY proteins (lane 11 in Figure 2). The most evident bands around 38–40 kDa on the electrophoretic pattern (line 11) were assigned to the incomplete digestion of α- and β-livetins from the EY. As expected, a lower hydrolysis efficiency was noticed in the case of the WE and the EW samples because of the serine proteases inhibitory activity exerted by various proteins originating from the EW, as discussed before. However, of particular importance is that the WE and the EW treatment with proteinase K resulted in a reduction in the intensity of the electrophoresis gel bands corresponding to OVA and OVM (lanes 11 and 13 in Figure 2). A small band around 40 kDa can be observed (Figure 2, lane 12), which was associated with an undigested part of OVA.

### 2.3. Influence of Enzyme-Assisted Hydrolysis on the Antioxidant Activity of the Egg Proteins

**Figure 3:**
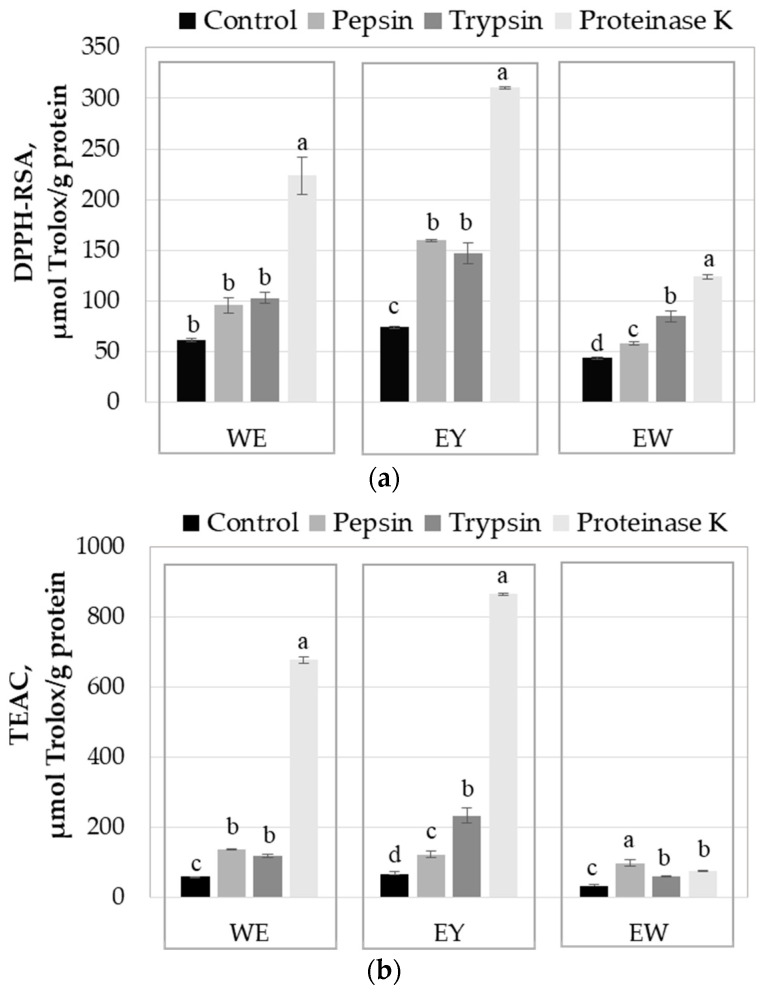
Influence of hydrolysis with pepsin, trypsin and proteinase K on the DPPH radical scavenging (DPPH-RSA) activity (**a**) and Trolox equivalent antioxidant capacity (TEAC) (**b**) of a whole egg (WE), egg yolk (EY) and egg white (EW). Controls were prepared with no enzyme addition. For an egg derivative (WE, EY and EW), different letters (a, b, c, d) assigned to the mean values indicate significant differences among enzyme treatments, at *p* < 0.05, based on Tukey post-hoc test, and 95% confidence.

The antioxidant activity of the egg products used in the study (WE, EY and EW) and of the protein hydrolysates obtained with pepsin, trypsin and proteinase K was determined using the DPPH-RSA and TEAC methods, and the results are presented in Figure 3. As expected, among all egg products, the EY exhibited the highest antioxidant activity, followed by the WE and the EW. Many compounds found in eggs, such as vitamin E, vitamin A, carotenoids, selenium, phospholipids and some proteins, exhibit antioxidant activity [26].

Regardless of the investigated egg protein derivative, both methods indicated that the enzyme-assisted hydrolysis resulted in a significant increase in antioxidant activity (Figure 3). Some properties of the egg proteins, beyond satisfying the nutritional requirements for growth, are assigned to the bioactive peptides encrypted in the parent proteins [8]. An important number of peptides are able to exert various biological activities only upon being released from the polypeptide chains. Among the biological activities with a positive impact on bodily functions and human health, the antioxidant activity of the peptides plays a central role. The food-derived peptides mainly exhibit their antioxidant activity thorough scavenging the free radicals or reactive species, the reaction mechanisms involving the H or proton-coupled single electron transfer [27]. Additional mechanisms involve inactivation of the reactive species, chelation of the metal ions with pro-oxidative properties and reducing the hydroperoxides [26].

The peptide length and amino acid composition are the main factors influencing the antioxidant properties. The low molecular weight peptides, with 2–20 amino acid residues, are known for their high antioxidant activity [26]. The increase in antioxidant activity after the enzyme-assisted hydrolysis of egg proteins (Figure 3) might be explained by the exposure of the electron-dense peptide bonds and of the functional side chains of the amino acids, which became prone to access the reactive species [26,27]. Regarding the antioxidant capacity of the free amino acids, Udenigwe and Aluko [27] indicated that the sulfur-containing (Cys and Met), acidic (Glu and Asp) and hydrophobic amino acids (Pro, Ala, Val, Ile, Leu, Tyr, Phe and Trp) had strong positive effects on DPPH scavenging. Moreover, Xu et al. [28] classified Trp, Met, His, Lys, Cys, Arg and Tyr, as the amino acids with the strongest total antioxidant capacity.

Analysing the results presented in Figure 3a, one can observe that, in the case of all the egg protein-based substrates, proteinase K released the peptides with stronger antioxidant activity. Although proteinase K is able to act on a broad range of peptide bonds, it has better specificity for those involving aromatic or aliphatic hydrophobic amino acids at the N-terminal. Therefore, it is expected to generate important amounts of peptides with highly active amino acids at the N-terminal, such as Tyr or Trp [28], which explains the high antioxidant activity registered for the egg protein hydrolysates obtained with proteinase K. The side chains of these amino acids consist of electron-rich aromatic rings, which are susceptible to attack by oxidants [28]. The presence of the hydroxyl group in Tyr residue located at the N-terminus of the peptides might contribute to an increase in the antioxidant activity. Moreover, Udenigwe and Aluko [27] stated that the antioxidant activity of the peptides is superior to the constituent amino acids.

The relationship between HD and the bioactivity of the resulting peptides mainly depends on the protein substrate and the enzymes used for hydrolysis. Although pepsin ensured a higher hydrolysis degree of the egg white proteins compared to trypsin and proteinase K, the peptides released by the latter enzymes exhibited better antioxidant activity (Figure 3). Trypsin is an intestinal digestive serine protease that breaks the peptide bonds of the Arg and Lys residues on the C-terminal side [29]. Because of the nitrogen atoms with one lone electron pair in the side chains, these amino acids can be attacked by oxidants [28]. Therefore, the peptides released by this enzyme will include one amino acid residue with strong antioxidant capacity at the C-terminal. Finally, pepsin preferentially breaks the peptide bonds involving one of the amino acids Phe, Tyr, Trp or Leu, with demonstrated antioxidant capacity [27,28].

Except for the EW-T and the EW-K samples, the antioxidant activity results obtained for the egg protein hydrolysates using the TEAC method were higher compared to those determined with the DPPH-RSA method. A similar trend was noticed by Dumitrașcu et al. [30], who investigated the antioxidant properties of the peptides obtained from spent brewer’s yeast and assigned the differences to the higher stability of the DPPH radical compared to ABTS+ and to the particularities of the reaction mechanisms of the two methods. The lower TEAC values measured for the EW-T and the EW-K samples might be explained by the release of particular peptides, which needed a longer time to reach the endpoint of the TEAC reaction [31].

A significant number of studies focused on testing different proteases for their ability to release bioactive peptides out of egg proteins. As reviewed by Benedé and Molina [26], pepsin, trypsin and chymotrypsin are the most commonly used enzymes reported to produce egg yolk and white hydrolysates with high antioxidant activity. Other commercial enzymes, including neutrase, papain, protamex, collupulin, ficin, flavourzyme, protease M and protease P, as well as microbial protease obtained through fermentation, proved to be effective in producing egg white hydrolysates with high antioxidant activity [26].

However, it should be noted that the antioxidant activity of the hydrolysates generated by a particular enzyme highly depends on the hydrolysis degree. Many enzymes are able to release various encrypted peptides in the early stages of the hydrolysis, which exhibit biological activities but complete or more advanced hydrolysis could lead to the loss of their functionality as a result of MW reduction. The complete hydrolysis of the main egg proteins by the enzymes used in the laboratory scale experiment was simulated by means of the bioinformatics approach, and the bioactivity of the resulting peptides was verified against the records from the BIOPEP-UWM database [27]. Analysing the results presented in Table 1, one can observe that mainly the di- and tri-peptides are responsible for the antioxidant properties of the hydrolysates when complete hydrolysis is achieved. Among all the investigated enzymes, proteinase K appears to release the highest amounts of di- (EL, HL, RW, KP, TY, AW and RY,), tri- (KAI, KKY, QAY, SDF and KAY,) and tetrapeptides (SGAF, ADGF) from the proteins originating both from egg white and yolk. A limited variety of dipeptides (EL and HL) result from the extensive hydrolysis of ovalbumin, ovotransferrin and mucin-6 with pepsin, whereas trypsin activity generates peptides with antioxidant activity (IR, LK, AHK, LHR and DYK) mainly when acting on vitellogenin-2 (Table 1).

### 2.4. Influence of Protein Hydrolysis on the Antigenic Properties of Egg and Egg Derivatives

In the absence of an efficient treatment, the strict avoidance of egg in the diet of the allergic subjects is the only manner to prevent any allergic reaction. However, Ma et al. [5] stated the importance of developing egg-derived products and ingredients with hypoallergenic properties. Because of the low affinity towards the IgE molecules, the probability of obtaining an in vivo anaphylactic response is significantly reduced in the case of hypoallergenic egg-based products [5].

It was shown previously that processing might exert a significant impact on the allergenic properties of egg proteins. The knowledge of the changes induced by different non-thermal and thermal processing techniques on the linear and/or conformational epitopes could be exploited for producing hypoallergenic egg products. In particular, enzyme-assisted hydrolysis of egg proteins was reported to be effective in reducing the allergenicity of different food allergens, as a consequence of breaking the peptide bonds within different lines of conformational epitopes.

**Figure 4:**
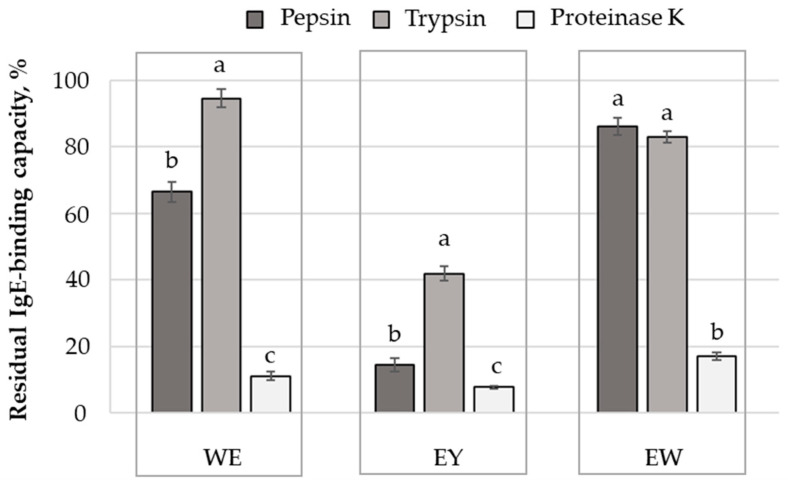
The residual IgE-binding capacity of the protein hydrolysates obtained through the extensive hydrolysis of the whole egg, egg yolk and egg white with pepsin, trypsin and proteinase K. For an egg derivative (WE, EY and EW), different letters (a, b, c) assigned to the mean values indicate significant differences among enzyme treatments, at *p* < 0.05, based on Tukey post-hoc test, and 95% confidence.

The ELISA technique was applied in the present study to estimate the impact of enzyme-assisted hydrolysis on the antigenic properties of egg proteins. Analysing the results presented in Figure 4, one can observe that all the investigated enzymes were able to decrease, to different extents, the antigenic properties of the egg derivatives. The most important IgE-binding capacity reduction was noticed in the case of egg yolk, which is known to contain minor allergens [5]. Previous studies indicated that the major egg allergens are located in egg whites. In particular, ovomucoid, ovalbumin, ovotransferin and lysozyme are responsible for the IgE-mediated reactions, also known as immediate hypersensitivity reactions [5,8]. The presence of high amounts of these proteins in egg whites and whole eggs might explain the significantly lower IgE-binding capacity reduction noticed when using these protein derivatives as substrates (Figure 4).

Regardless of the substrate subjected to hydrolysis, proteinase K was the most efficient in recognising the epitopes of the egg allergens, resulting in the lowest values (*p* < 0.05) of the residual IgE-binding capacity. The lowest efficiency in reducing the antigenicity of the egg proteins was noticed in the case of trypsin; the total IgE-binding capacity was lowered by ~5.4% in the case of the whole egg, by ~58.1% in the case of the egg yolk and by ~17.0% in the case of the egg white (Figure 4). The high residual IgE-binding capacity noticed in the case of the whole egg and egg white substrates are most likely due to the strong inhibitory trypsin activity exerted by OVM, which was incriminated for limiting the protein digestion and nutrient absorption in humans and animals [32]. The OVM is characterised by high thermal and chemical stability, which was mainly assigned to the existence of three disulfide bonds within each subdomain of the molecule, making it difficult to eliminate the trypsin inhibitory activity [32]. 

Regarding the performance of the pepsin to reduce the antigenic properties of the egg proteins, it can be observed that the susceptibility of the allergen proteins to hydrolysis was higher in the WE-P (residual IgE-binding capacity of 66.5%) compared to the EW-P sample (residual IgE-binding capacity of 86.2%). As in the case of our experiment (Figure 1), when simulating the gastric and duodenal digestion, Martos et al. [33] noticed that the egg white proteins were better hydrolysed in the presence of egg yolk. Nonetheless, no important differences in terms of immunoreactivity against IgE were registered. The authors assigned the residual antigenicity partially to some IgE-binding peptides originating from OVA, but also to the presence of intact OVA and lysozyme. Previous studies report on the ability of the enzymes to reduce the IgE reactivity of particular egg proteins. When assessing the digestibility of the ovalbumin with human digestive fluids, Benedé et al. [34] reported a lower IgE-binding ability of the resulting peptides. Moreover, Matsuda et al. [35] indicated the ability of pepsin to hydrolyse the ovomucoid into three major peptides with a MW of 25 kDa, 18 kDa and 13 kDa, which endured a remarkable decrease in in vivo allergenicity.

The allergenicity of egg proteins was generally related to their heat stability, their resistance to enzymatic digestion and the exposure of the existent IgE-binding epitopes [18]. In fact, the high prevalence of egg allergy in the case of infants and patients with incomplete maturation of the stomach function was assigned to their low pepsin activity, due to the low amounts of available enzymes or the pH values outside the optimum range of 1.5–2.0 [18]. Therefore, the efficient hydrolysis of the egg proteins by the enzymes of the gastrointestinal tract is highly desired because, in addition to ensuring the proper digestion and absorption processes, it allows for a reduction in the probability of the epitopes’ recognition by IgE. Factoring the limited retention time of the digesta in the segments of the digestive tract, such as the stomach and the small intestine where the main digestive proteases act, and also considering the trypsin inhibitory potential of various molecules from the substrate, the preliminary treatment of egg proteins with exogenous enzymes used as ingredients in food production is appealing. However, it should be taken into account the fact that enzyme-assisted hydrolysis of egg proteins could expose hidden epitopes, originally buried within the hydrophobic core of the native allergen proteins.

### 2.5. In Silico Observations of the Main Egg Allergens

**Figure 5:**
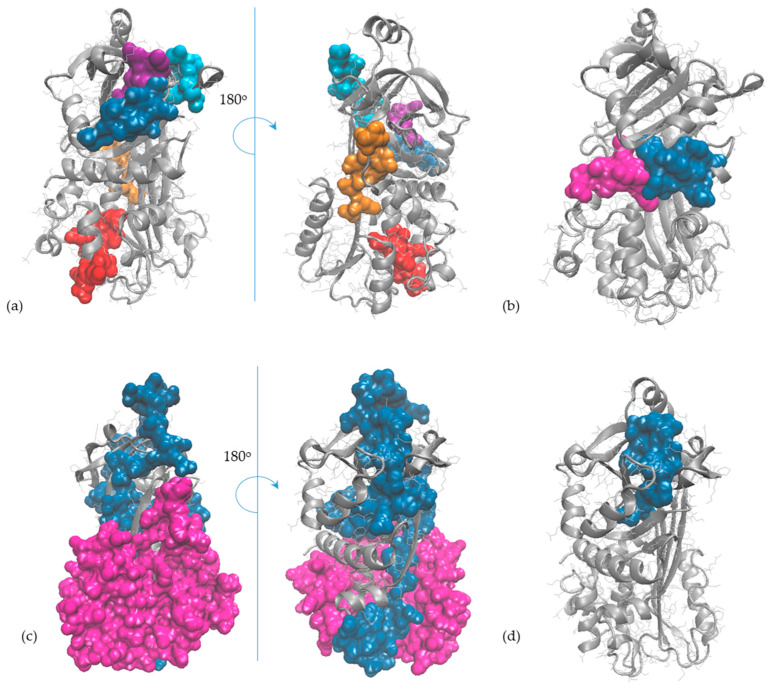
The IgE-binding epitopes of ovalbumin (represented in grey in New Cartoon style) reported in human patients by (**a**) Mine and Rupa [36] (the epitopes (39–50), (96–103), (192–201), (244–249) and (252–261) are represented in red, orange, cyan, purple and blue, respectively), (**b**) Elsayed et al. [37] (the (2–11) epitope is represented in magenta) and Elsayed and Stavseng [38] (the (12–20) epitope is represented in blue); (**c**) Kahlert et al. [39] (the epitopes (42–173) and (302–386) are represented in magenta and blue, respectively); (**d**) Honma et al. [40] (the (358–367) epitope is represented in blue).

**Figure 6:**
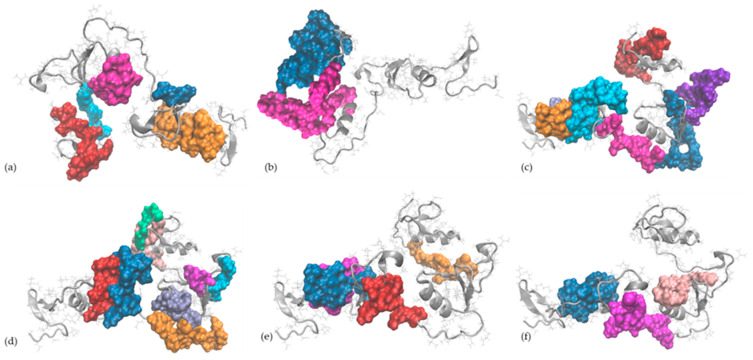
The IgE-binding epitopes of ovomucoid (represented in grey in New Cartoon style) reported by (**a**) Cooke and Sampson [42] (the epitopes (25–44), (73–74), (109–120), (139–146) and (175–186) are represented in orange, blue, magenta, cyan and red, respectively), (**b**) Besler et al. [43] (the epitopes (114–145) and (158–210) are represented in magenta and blue, respectively); (**c**) Holen et al. [44] (the epitopes (25–38), (35–48), (55–68), (75–88), (85–98), (125–138) and (145–158) are represented in orange, ice blue, cyan, magenta, blue, violet and red, respectively); (**d**) Mine and Zhang [45] (the epitopes (55–66), (64–74), (80–90), (95–99), (104–114), (125–129), (145–154) and (183–198) are represented in red, blue orange, magenta, ice blue, cyan, pink and green, respectively); (**e**) Jarvinen [46] (the epitopes (25–34), (35–44), (71–80) and (137–146) are represented in blue, magenta, red and orange, respectively); (**f**) Martínez-Botas et al. [41] (the epitopes (28–44), (70–83) and (115–128) are represented in blue, magenta and pink, respectively).

Molecular modelling and bioinformatics tools were further employed to gather information at the single-molecule level on ovalbumin and ovomucoid, the egg allergens recognised by the specific antibodies of the ELISA test used in this study (as indicated by the producer of RIDASCREEN^®^FAST Ei/Egg Protein kit). The careful analysis of the 3D models of OVA and OVM molecules indicated that all linear IgE-binding epitopes reported in the literature have good exposure to the protein’s surface (Figure 5 and Figure 6) being, therefore, suitable for enzyme-assisted hydrolysis, even when limited or moderate DH is achieved.

The IgE-binding epitopes of OVA and OVM, as reviewed by Mine and Yang [10] and Martínez-Botas et al. [41], are presented in Table 2. In order to check how enzyme-assisted hydrolysis can affect the integrity of the epitopes, the PetideCutter tool was further used to predict the cleavage sites on the OVA and OVM structures. Analysing the results presented in Table 2, one can observe that proteinase K appeared to be the most effective in targeting multiple peptides bonds within each linear epitope of the OVA and the OVM molecules. The only exception concerns the KRHDGGCRKE (145–154) epitope on OVM, which cannot be altered by this enzyme. These observations are in good agreement with the experimental results, showing that, among all the tested enzymes, proteinase K ensured the lowest residual IgE-binding capacity results for the egg white (Figure 4). At least one peptide bond of each IgE-binding epitope of OVA molecules was susceptible to hydrolysis with pepsin, but several epitopes located on domains II and III of the OVM molecule remained unaffected. In the case of both OVA and OVM, trypsin seems the least effective in recognising and breaking the peptide bonds within the IgE-binding epitopes (Table 2). These observations support the experimental results regarding the effect of enzyme-assisted hydrolysis on the residual antigenicity of proteins originating from egg whites (Figure 4).

## 3. Materials and Methods

### 3.1. Materials

The following powdered egg-based products produced by OVOSTAR LTD (Kyiv, Ukraine) were used in the study: whole eggs (Ovomix P5001 LF; 48.1% proteins, 43.9% fats and 5.0% moisture), egg yolk (P6003; 35.4% proteins, 51.0% fats and 4.0% moisture) and desugarised egg white (Ovomix P4000H; 82.2% proteins, 0.04% fats and 8.0% moisture). As indicated by the producer, the powdered chicken egg-based products were obtained through pasteurisation and spray drying. The hydrolysis of the egg proteins was performed using the following enzymes purchased from Merck (Darmstadt, Germany): pepsin (EC 3.4.23.1) from porcine gastric mucosa (0.7 FIP-U/mg), trypsin (3.4.21.4.) (0.2 FIP-U/mg) and proteinase K (EC 3.4.21.64) from Tritirachium album (30 mAnsonU/mg).

Ovomucoid, ovalbumin (purity over 90%), methanol (HPLC grade), 2,2-diphenyl-1-picrylhydrazyl (DPPH), 6-hydroxy-2,5,7,8-tetramethylchroman-2-carboxylic acid (Trolox), 2,2′-azino-bis (3-ethylbenzothiazoline-6-sulfonic acid) diammonium salt (ABTS), sodium dodecyl sulphate (SDS) were purchased from Sigma-Aldrich Chemie GmbH (Taufkirchen, Germany). All other chemicals and solvents were of analytical grade.

### 3.2. Obtaining the Egg Protein Hydrolysates

The whole eggs powder, egg white powder and egg yolk powder were used to prepare suspensions of 6% (*w/v*) protein concentration in distilled water. In order to ensure the appropriate hydration of the powders, the mixtures were homogenised for 2 h at temperature of 20 ± 1 °C by using a magnetic stirrer, and were afterwards stored overnight under refrigeration conditions (4 ± 1 °C). Prior to enzyme addition, the suspensions were tempered at 37 °C, and the pH was adjusted to 2.0 in the case of the samples hydrolysed with pepsin (samples codded WE-P, EW-P and EY-P, respectively), and to 8.0 in the case of the samples hydrolysed with trypsin (samples codded WE-T, EW-T and EY-T, respectively) or proteinase K (samples codded WE-K, EW-K and EY-K, respectively), using NaOH or HCl of appropriate concentration. The enzyme/substrate ratio of 1: 100 (*w/w*) was used, and the hydrolysis was allowed to occur for 24 h at 37 °C. The enzyme was afterwards inactivated by heating for 20 min at 90 °C. Upon cooling to room temperature all hydrolysates were subjected to freeze drying (CHRIST Alpha 1-4 LD plus, Osterode am Harz Germany). Parallel control samples were prepared without enzyme addition.

### 3.3. Determination of the Degree of Hydrolysis

The DH was determined using the pH-stat method, as described by Salelles et al. [47]. The volume (V, mL) of acidic titrant (HCl), in the case of the egg protein-based samples hydrolysed with pepsin at pH 2.0, or basic titrant (NaOH), in the case of the samples hydrolysed with trypsin or proteinase K at pH 8.0, was used to calculate the DH (%) using Equations (1) and (2), respectively:

(1) $$DH=\frac{V\times N}{m\times h_{tot}\times (1-\alpha_{COOH/COO^{-}})}\times 100$$

(2) $$DH=\frac{V\times N}{m\times h_{tot}\times \alpha_{{NH}_{3}^{+}/{NH}_{2}}}\times 100$$

where N is the normality of the acidic or basic titrant, m is the mass of protein sample (g), h_tot_ = 8.0 meqv/g and represents the total number of peptide bonds per gram of protein, $\alpha_{COOH/COO^{-}}$ = 0.02 and represents the average dissociation degree of the carboxylic groups of the peptides resulting from hydrolysis [47], while $\alpha_{{NH}_{3}^{+}/{NH}_{2}}$ = 0.79 and represents the average dissociation degree of amine groups of the peptides resulting from hydrolysis [48].

### 3.4. SDS-PAGE Analysis

The molecular weight distribution in the whole egg, egg yolk and egg white samples subjected to hydrolysis with pepsin, trypsin and proteinase K, was evaluated by sodium dodecyl sulfate−polyacrylamide gel electrophoresis (SDS-PAGE). The protein samples were diluted with Laemmli sample buffer 2x (Serva, GmbH, Heidelberg, Germany), treated with 1 M β-mercaptoethanol to create reduction conditions and, then heated at 95 °C for 5 min before loading onto the 4.5% acrylamide of the stacking gel (pH 6.8) [49]. In the case of the resolving gel, a concentration of 12% acrylamide (pH 8.8) was made using 30% acrylamide/bis-acrylamide in a ratio of 37.5:1, with a 2.6% crosslinker (Bio-Rad, Hercules, CA, USA). The electrophoretic separation was achieved in running buffer (Bio-Rad, Hercules, CA, USA) at a constant voltage of 100 V for 90 min. The gels were fixed in 40% methanol:10% acetic acid (*v/v*), stained for 40 min in 0.1% *w/v* Coomassie Brilliant Blue R-250 (Sigma-Aldrich, Saint Louis, MO, USA) and de-stained in 10% *v/v* acetic acid. The Precision Plus Proteins Dual Xtra Prestained marker (Bio-Rad, Hercules, CA, USA) was loaded into each gel, together with the commercial ovalbumin (OVA) and ovomucoid (OVM), which were prepared in the same way as the samples.

### 3.5. Antioxidant Activity 

The antioxidant activity of the egg protein hydrolysates was measured using the DPPH radicals scavenging activity (DPPH-RSA) and Trolox equivalent antioxidant capacity (TEAC) methods, as described by Banu et al. [50]. 

The DPPH-RSA was determined by mixing 0.1 mL of sample with 0.25 mL of DPPH solution (10 mg DPPH in 25 mL 80% methanol) and 2 mL of 80% methanol, followed by incubation for 20 min under dark conditions and absorbance measurement at 515 nm. The blank was prepared by using 0.1 mL of 80% methanol instead of samples.

For the ABTS-RSA method, a volume of 1.48 mL of fresh ABTS·+ solution (with an absorbance of 0.700 ± 0.030 at 734 nm) was added to 20 μL of sample and the absorbance at wavelength of 734 nm of the reaction mixture was measured immediately and after 6 min of incubation.

In the case of both methods, the results were reported as µM Trolox Equivalent (TE)/g protein.

### 3.6. Assessment of the Bioactive Peptides with Antioxidant Activity

The peptides with potential antioxidant activity released through complete hydrolysis of the main egg proteins with pepsin, trypsin and proteinase K were further identified by means of the bioinformatics tools. The records on the primary structure of the proteins were taken from the UniProt database [51], as follows: P01012 (Ovalbumin), P02789 (Ovotransferrin), P01005 (Ovomucoid), P00698 (Lysozyme), Q98UI9 (Ovomucin α subunit) and F1NBL0 (Ovomucin β subunit) are the codes for the main egg white proteins, whereas P87498 and P02845 (Vitellogenin-1 and Vitellogenin-2 which are cleaved into lipovitellin-1, phosvitin, lipovitellin-2 and YGP40), and P19121 (α Livetin) are the codes for the main proteins from the egg yolk. The BIOPEP-UWM database [52] was further used to check the ability of pepsin, trypsin and proteinase K to release the bioactive peptides with antioxidant activity, originally encrypted in the main egg proteins.

### 3.7. IgE-Binding Properties

The enzyme-linked immunosorbent assay (ELISA) was used to assess the impact of enzyme hydrolysis on the antigenic properties of egg proteins. The RIDASCREEN^®^FAST Ei/Egg Protein kit (Biopharm AG, Darmstadt, Germany) was selected for the rapid quantitative determination and a Stat Fax 4200 microplate reader (Awareness Technology, Palm City, FL, USA) was used to measure the absorbance. All samples were appropriately diluted before running the ELISA protocol. The NIST reference material included in the kit was used to perform the calibration. The residual IgE-binding capacity of the egg proteins upon applying the enzyme-assisted hydrolysis treatment was calculated, in agreement with Stănciuc et al. [11], as percentage of allergen protein detected by the specific antibodies with respect to the egg protein derivative used as substrate for proteolysis. Duplicate measurements were performed for each replicate sample.

### 3.8. In Silico Investigations on the Main Egg Allergens

Single-molecule level investigations on the major egg allergens, ovalbumin and ovomucoid, were carried out by means of bioinformatics and molecular modelling tools. The molecular model of ovalbumin (OVA) was obtained from the RSCB Protein Data Bank (1OVA.pdb, [53]), whereas the 3D model of ovomucoid (OVM) was predicted starting from P01005 entry in UniProtKB database [51] using the I-TASSER algorithm [54,55]. The protein models were prepared for the molecular dynamics production step by first running molecular mechanics steps meant to optimise the solvated molecules. The OVA and OVM models were heated and equilibrated using Gromacs 4.6.1 package, as indicated in Stănciuc et al. [11]. The final models were checked for the exposure of the linear epitopes, and the PeptideCutter tool (ExPASy server; https://www.expasy.org/ (accessed on 10 January 2024)) was used to check the on each investigated egg allergen the cleavage sites recognised by pepsin, trypsin and proteinase K.

### 3.9. Statistical Analysis

The experimental results were expressed as mean ± standard deviation values. The statistical analysis using one-way ANOVA and Tukey test was carried out using Minitab 19 (Minitab LLC, State College, PA, USA) software. The statistical differences between samples were indicated at *p* < 0.05.

## 4. Conclusions

The antioxidant properties and IgE-binding ability of the protein hydrolysates obtained from whole eggs, yolks and whites using pepsin, trypsin and proteinase K were investigated. In the case of all the studied substrates, the peptides released by pepsin, trypsin and proteinase K exhibited substantially stronger antioxidant properties compared to the parent egg proteins. The functional peptides released by the enzymes were inactive when encrypted in the parent proteins. The highest DPPH radical scavenging activity values were registered for the hydrolysates obtained with proteinase K.

Among all the studied enzymes, proteinase K allowed for the most significant reduction of an egg protein’s antigenic properties. The molecular modelling investigations carried out on the main egg allergens, revealed that the enzyme might ensure the allergenicity reduction by destroying the major linear epitopes with high exposure to the surface of ovalbumin and ovomucoid molecules. Therefore, proteinase K might be a suitable biotechnological tool for producing egg protein-based ingredients with reduced antigenic properties and improved antioxidant capacity. However, it should be noted that, in addition to the applied processing technique and selected parameters, the allergenicity of the egg proteins might also be influenced by the food matrix. Therefore, further investigations should concern the elucidation of the influence of the processing of the egg ingredients included in different food formulations on the antigenic properties of the proteins. Moreover, the effect of other endopeptidases that are generally recognised as safe might be tested to identify the most promising enzymes for improving the intrinsic properties of the proteins from whole eggs, yolks and whites.

## Figures and Tables

**Table 1:** Potential peptides with antioxidant activity released after complete hydrolysis with pepsin, trypsin and proteinase K of the main proteins prevailing in egg white and egg yolk. The complete hydrolysis of egg proteins was simulated by means of the bioinformatics approach, and the bioactivity of the resulting peptides was predicted by checking the content of the BIOPEP-UWM database.

Protein Name (UniProt Entry)	Total No. of Encrypted Peptides with Antioxidant Activity	Potential Peptides with Antioxidant Activity Released through Complete Hydrolysis with Various Enzymes		
		Pepsin	Trypsin	Proteinase K
Egg white				
Ovalbumin (P01012)	38	EL	-	EL
Ovotransferrin (P02789)	75	EL, HL	-	EL, HL, RW, KP, KAI, SGAF
Ovomucoid (P01005)	14	-	-	KP, TY
Lysozyme (P00698)	15	-	-	AW
Ovomucin α subunit (Q98UI9)	142	-	IR	EL, KP, RY, SDF
Ovomucin β subunit (F1NBL0)	76	EL, HL	-	EL, HL, KKY, QAY
Egg yolk				
Vitellogenin-2 (P02845)	149	HL, VKL	IR, LK, AHK, LHR, DYK	EL, HL, AY, KP, RY, TY, AW, KAY
Vitellogenin-1 (P87498)	126	EL	IR	EL, HL, AY, KP, RY, RW, TY
α Livetin (P19121)	31	-	-	RY, ADGF

**Table 2:** Prediction of the susceptibility to enzyme-assisted hydrolysis of the IgE-binding epitopes of ovalbumin (UniProt ID P01012) and ovomucoid (UniProt ID P01005). The cleavage sites recognised by pepsin, trypsin and proteinase K were identified upon simulating the complete hydrolysis of the proteins using the PeptideCutter tool.

Ref.	Amino Acid Sequence [Position in P01012/P01005]	Cleavage Sites		
		Pepsin	Trypsin	Proteinase K
Ovalbumin				
Mine and Rupa [36]	LAMVYLGAKDST (39–50)	39 43 44	47	39 40 42 43 44 46
	DVYSFSLA (96–103)	99 100 101 102	105	97 98 100 102
	EDTQAMPFRV (192–201)	198	200	192 194 196 199
	VLLPDE (244–249)	244 246	-	224 226 230 231 232 233 235 236 239 243 244 245 246
	GLEQLESIIN (252–261)	252 253 255 256	-	253 254 256 257 259 260
Elsayed et al. [37]	GSIGAASMEF (2–11)	10	-	4 6 7 10
Elsayed and Stavseng [38]	CFDVFKELK (12–20)	12 13 15 16 18	-	13 15 16 18 19
Kahlert et al. [39]	VYLGAKDSTRTQINK VVRFDKLPGFGDSIE AQCGTSVNVHSSLR DILNQITKPNDVYSF SLASRLYAEERYPILP EYLQCVKELYRGGLE PINFQTAADQAREL INSWVESQTNGIIRN VLQPSSVDSQTAM (42–173)	43 44 60 63 66 84 88 99 100 101 102 106 115 118 119 124 134 135 144	47 51 56 59 62 85 105 111 123 127 143 159	42 43 44 46 50 52 54 57 58 60 63 66 70 71 72 76 78 80 84 87 88 91 92 97 98 100 102 103 106 107 108 109 110 112 114 115 117 118 119 122 124 125 126 130 131 133 135 137 138 139 142 144 145 146 149 150 151 154 157 158 161 162 167 171
	GITDVFSSSANLSGI SSAESLKISQAVHA AHAEINEAGREVV GSAEAGVDAASVS EEFRADHPFLFCIK HIATNAVLFF GRC VSP (302–386)	306 307 312 313 321 322 358 359 364 366 367 377 378 379 380	323 340 360 370 382	304 306 307 311 313 316 319 320 322 324 327 328 330 331 333 334 335 337 338 341 342 343 346 347 348 350 352 353 355 357 358 359 361 365 366 367 369 372 373 374 376 377 378 379 380 384
Honma et al. [40]	EFRADHPFLF (358–367)	358 359 364 366	360	358 359 361 365 366
Ovomucoid				
Cooke and Sampson [42]	AEVDCSRFPNATDKEGKDVL [25,26,27,28,29,30,31,32,33,34,35,36,37,38,39,40,41,42,43,44]	32	31 38 41	25 26 27 32 35 36 39 43
	EF (73–74)	73	-	73
	VLCNRAFNPVCG (109–120)	109 110 114	113	109 110 114 115 118
	QGASVDKR (139–146)	-	145	141 143
	RPLCGSDNKTYG (175–186)	176	-	177 184 185
Besler et al. [43]	AFNPVCGTDGVTYDNECLLCAHKVEQGASVDK (114–145)	114 130 131 132	136 145	114 115 118 121 124 125 126 129 131 132 134 137 138 141 143
	VSVDCSEYPKPDCTAEDRPLCGSDNKTYGNKCNFCNAVVESNGTLTLSHFGKC (158–210)	176 191 201 202 203 204 206 207	183 188 209	158 160 164 165 171 172 173 177 184 185 191 194 195 196 197 201 202 203 204 207
Holen et al. [44]	AEVDCSRFPNATDK (25–38)	32	31	25 26 27 32 35 36
	ATDKEGKDVLVCNK (35–48)	32 44	38 41	35 36 39 43 44 45
	GTDGVTYTNDCLLC (55–68)	65 66 67		56 59 60 61 62 66 67
	GTNISKEHDGECKE (75–88)	-	80 87	76 78 81 85
	ECKETVPMNCSSYA (85–98)	-	87	85 88 89 90 97
	TYDNECLLCAHKVE (125–138)	130 131 132	136	125 126 129 131 132 134 137
	KRHDGGCRKELAAV (145–158)	-	145 146 153	143 154 155 156 157
Mine and Zhang [45]	TDGVTYTNDCL (56–66)	65	-	56 59 60 61 62
	DCLLCAYSIEF (64–74)	65 66 67 73	-	66 67 69 70 72 73
	KEHDGECKETV (80–90)	-	80 87	81 85 88 89
	SSYAN (95–99)	-	-	97 98
	DGKVMVLCNRA (104–114)	109 110	106 113	107 109 110
	TYDNE (125–129)	-	-	125 126
	KRHDGGCRKE (145–154)	-	145 146 153	-
	KTYGNKCNFCNAVVES (183–198)	191	183 188	184 185 191 194 195 196 197
	TLSHFGKC (203–210)	203 204 206 207	209	203 204 207
Jarvinen [46]	AEVDCSRFPN (25–34)	32	31	25 26 27 32
	ATDKEGKDVL (35–44)	32	38 41	35 36 39 43
	SIEFGTNISK (71–80)	73 74	-	70 72 73 74 76 78
	VEQGASVDKR (137–146)	-	145	137 138 141 143
Martínez-Botas et al. [41]	DCSRFPNATDKEGKDVL (28–44)	32	31 38 41	32 35 36 39 43
	YSIEFGTNISKEHD (70–83)	73 74	80	70 72 73 74 76 78 81
	FNPVCGTDGVTYDN (115–128)	-	-	115 118 121 124 125 126

## Data Availability

Data are contained within the article.
